# Strategies to Overcome Hematocrit and Volume Bias in Dried Blood Spot Analysis

**DOI:** 10.3390/ph19030403

**Published:** 2026-03-01

**Authors:** Panagiotis-Dimitrios Mingas, Matjaž Cirar, Iztok Grabnar, David Drobne, Tomaž Vovk

**Affiliations:** 1 Faculty of Pharmacy, University of Ljubljana, 1000 Ljubljana, Slovenia; panagiotis-dimitrios.mingas@ffa.uni-lj.si (P.-D.M.); iztok.grabnar@ffa.uni-lj.si (I.G.); 2 Department of Gastroenterology, University Medical Centre Ljubljana, 1000 Ljubljana, Slovenia; david.drobne@kclj.si; 3 Department of Internal Medicine, Faculty of Medicine, University of Ljubljana, 1000 Ljubljana, Slovenia

**Keywords:** dried blood spots, DBS, microsampling, blood volume, hematocrit, image analysis, conductivity, spectrometry, bias

## Abstract

**Background/Objectives**: Dried blood spot (DBS) sampling, a technique for collecting capillary blood samples, is widely used in therapeutic drug monitoring, pharmacokinetic and toxicology research, newborn screening, and population health because it enables simple, non-invasive sampling across large cohorts. However, it presents several challenges, mainly due to the effect of hematocrit (HCT), which can influence the quantification of analytes. **Methods**: A combination of methods was developed to estimate the HCT and blood volume in DBS samples. Image analysis and hemoglobin (Hb) quantification using UV-VIS spectrometry were used for HCT estimation, and conductivity was used to determine blood volume. DBS samples from five donors were prepared with HCT between 0.2 and 0.6 and were used to prepare calibrators and quality control samples. The developed methods were applied to 23 samples obtained from ten adult patients with inflammatory bowel disease. **Results**: The methods for HCT determination using image analysis or Hb measurements were linear (r^2^ > 0.994), with acceptable accuracy (90.3–102.2%) and precision (<7.4%). Moreover, the conductivity method was linear (r^2^ = 0.999) and enabled accurate (96.8–100%) and precise (<5.65%) determination of blood volume in DBS samples. All three methods were in good agreement with the reference values in patient samples. Finally, strategies to correct HCT- and volume-related bias in DBS samples were proposed for analytes with different blood cell-to-plasma partition coefficients. **Conclusions**: We accurately and precisely estimated HCT in DBS samples using image analysis and Hb determination, and the volume of blood in DBS using conductivity measurement. We evaluated different approaches and derived an optimal procedure for HCT-bias correction.

## 1. Introduction

Dried Blood Spot (DBS) sampling is a minimally invasive microsampling technique used to collect and analyze small volumes of blood for biochemical and clinical purposes. It involves collecting a small amount of capillary blood, obtained via a fingertip or heel prick, which is then spotted onto an absorbent paper card. After the sample dries, the DBS paper card is ready for transportation or storage. One of the earliest applications of DBS was in newborn screening for various diseases. It has also been used for chronic disease monitoring (e.g., diabetes glucose testing) and infectious disease testing (e.g., hepatitis), especially in areas with limited healthcare infrastructure [1,2,3]. More recently, DBS has been used for the detection of coronavirus disease 2019 [4]. DBS plays an important role in therapeutic drug monitoring (TDM), where the concentrations of specific medications (e.g., anti-epileptics) are monitored, and therapy can be optimized accordingly [5]. Additionally, DBS is widely used in pharmacokinetic and toxicology research and epidemiological studies because it allows easy, non-invasive sampling across large cohorts.

The main advantages of this method are its simplicity and practicality. DBS samples are lightweight, small, and stable at ambient temperature. It is a minimally invasive sampling method that requires only a small amount of blood, thereby reducing patient discomfort. It is also cost-effective because storage and shipping costs are lower compared to traditional biosampling [6]. However, the application of the DBS method presents several challenges and limitations. The small sample volume limits the number and type of analyses performed. Sample heterogeneity is an additional challenge, as uneven spreading may lead to inconsistent results. Appropriate sample handling and storage to prevent contamination or degradation must be considered during method development.

The most critical source of variability in the DBS method is the hematocrit (HCT) bias, which should be systematically assessed. Addressing HCT-related variability is challenging, as its effect may vary depending on the compound analyzed, affecting the analytical results and causing significant assay bias at extreme HCT values. HCT bias can be divided into HCT-based area bias, HCT-based recovery bias, and HCT-based matrix effect bias. The most frequently reported bias, the HCT-based area (or volume) bias, arises from differences in blood viscosity, which affect the adsorption of blood on the filter paper and the spot size, potentially impacting the accuracy and quality of test results [7,8,9,10]. One approach to mitigate bias is to analyze the entire volumetrically applied DBS sample, instead of using a DBS punch. However, this method excludes the direct spotting of blood from the fingertip onto the paper card and may pose challenges in home sampling. In this case, the blood volume could be estimated without the need for HCT measurement, and the analytical results could be corrected based on the volume of blood on the DBS punch. Such methods attempt to avoid HCT bias; alternatively, estimating the HCT directly from DBS samples may offer a viable correction strategy. Multiple methods have been developed to determine HCT directly from DBS samples, providing a correction for the bias caused by varying HCT values [7,10].

This study aimed to compare methods that can potentially address the issues caused by the HCT effect, which may influence analyte quantification. These methods may assist in determining either the blood volume or HCT of a DBS sample when one of these parameters remains unknown. To address these issues, we compared methods that allow the estimation of both blood volume and HCT in DBS samples. During method development, we considered the practical obstacles encountered daily in healthcare facilities, such as limited sample availability.

## 2. Results

### 2.1. Hematocrit Determination in DBS Samples

#### 2.1.1. Hematocrit and Surface Area Measurements Using Image Analysis

**Figure 1:**
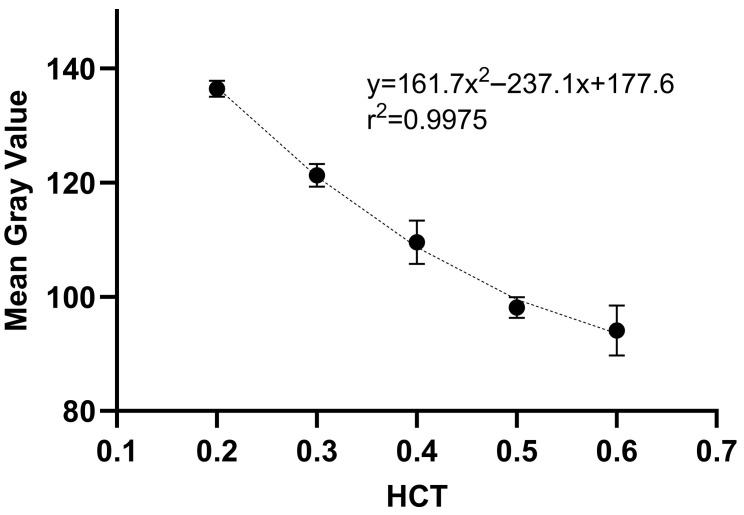
Polynomial relationship between HCT and MGV.

**Figure 2:**
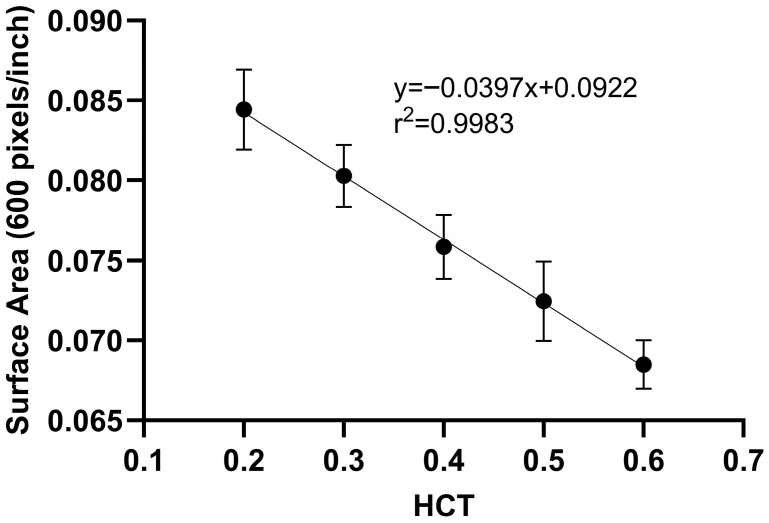
Linear relationship between HCT and DBS surface for volumetrically applied 20 μL DBS.

The summarized results of the DBS image analysis using Fiji software are presented in Table 1 and Table 2. The MGV and area of DBS in relation to HCT (0.2–0.6) are shown for 20 μL DBS, which was prepared volumetrically. A second-order polynomial relationship (Figure 1) between HCT and MGV was identified, and a linear relationship between HCT and DBS surface area was established (Figure 2). The high r^2^ values indicated good agreement between the predicted and nominal HCT values, demonstrating the suitability and reproducibility of the image analysis methodology using the Fiji software. As shown in Figure 1, as the HCT increases, the MGV decreases. In general, DBS samples with higher HCT (0.5, 0.6) appear much darker compared to DBS samples with lower HCT (0.2, 0.3). Similarly, as shown in Figure 2, the area of a 20 μL DBS decreased with increasing HCT values. This is related to the increase in blood viscosity with higher HCT, resulting in variations in the spreading on filter paper. These findings highlight the importance of considering HCT when interpreting DBS-based assays. For both methods, the accuracy ranged from 90.3% to 103%, while the precision did not exceed 11.3% (Table 1 and Table 2).

**Figure 3:**
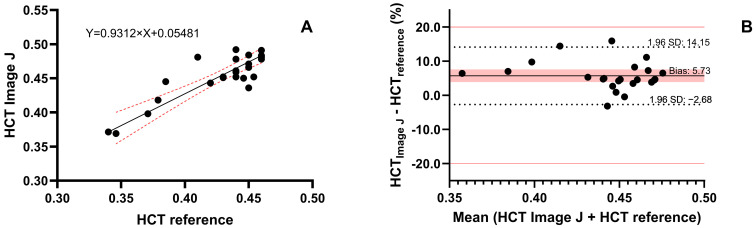
Deming regression (**A**) and Bland–Altman analysis (**B**) of HCT in patient samples. HCT values were determined in fresh blood using the reference method and in DBS samples using image analysis. Deming regression shows a linear regression line as a solid black line, with red dotted curves indicating the 95% confidence interval. In the Bland–Altman plot, the bias is shown as a solid black line, and the red area indicates the 95% confidence interval. The limits of the 95% interval of agreement are indicated by dotted black lines, whereas a deviation of 20% is indicated by solid red lines.

The image analysis method was used to determine the HCT in 23 samples from 10 patients, and the results were compared with the reference HCT values (Figure 3). The agreement of the methods was evaluated using Deming regression, which yielded a slope of 0.93 (95% confidence interval (CI) 0.71 to 1.15), and an intercept of 0.055 (95% CI: −0.041 to 0.151). Additionally, the Bland–Altman analysis showed good agreement with the reference method, as all samples deviated by less than 20%. The analysis yielded an MPPE of 4.88% and MAPE of 4.88%, indicating a consistent systematic bias between the image-based and reference HCT measurements. However, this difference is likely to be clinically irrelevant.

#### 2.1.2. Determination of HCT by Measuring Hemoglobin

**Figure 4:**
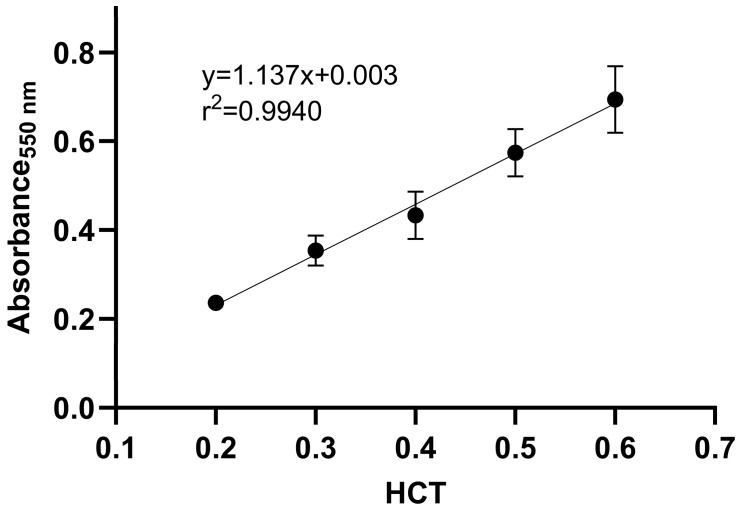
Linear relationship between HCT and absorbance at 550 nm.

Calibrators and quality control samples obtained from five donors at five HCT levels (0.2, 0.3, 0.4, 0.5, and 0.6) were analyzed. The results showed a linear relationship (r^2^ = 0.994) between HCT and the absorbance (Figure 4). Using the calibration curve, the HCT of the DBS sample can be determined after measuring the sample’s absorbance. Table 3 shows the RSD (<10%) and accuracy (within ±5% error) for the QC samples, which meet the recommended limits.

**Figure 5:**
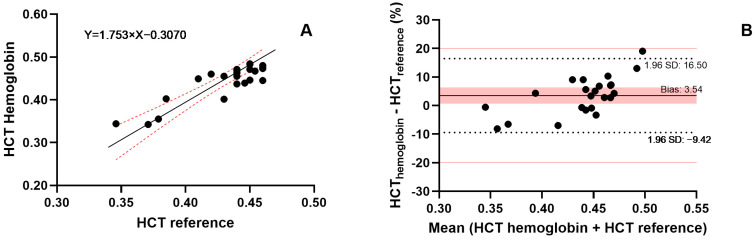
Deming regression (**A**) and Bland–Altman analysis (**B**) of HCT in patient samples. HCT values were determined in fresh blood using the reference method and in DBS samples using the hemoglobin method. The Deming regression shows a linear regression line as a solid black line, with red dotted curves indicating the 95% confidence interval. In the Bland–Altman plot, bias is shown as a solid black line, and the red area indicates the 95% confidence interval. The limits of the 95% interval of agreement are indicated by dotted black lines, while a deviation of 20% is indicated by solid red lines.

HCT was estimated using Hb measurements in 23 samples from 10 patients (Figure 5) and compared to reference HCT values obtained with a hematology analyzer. Deming regression yielded a slope of 1.75 (95% CI: 1.04–2.47) and an intercept of −0.31 (95% CI: −0.62 − 0.002). While the slope indicates a statistically significant proportional bias (95% CI excludes 1), the 95% CI of the intercept includes zero, suggesting no significant additive bias.

Importantly, possible overestimation of the HCT at high HCT values resulting from the bias is attenuated by the negative intercept within the HCT range of patients included in this study (0.35–0.46). This is also observed in the Bland–Altman analysis, which shows good agreement with the reference method, as all samples deviated by less than 20%. However, a clear trend of underprediction at low HCT values and overprediction at high HCT values is evident in the Bland–Altman plot (Figure 5B). An MPPE of 3.41% and MAPE of 4.67% suggest that HCT measurements using the Hb method are comparable to those from the reference method and are clinically acceptable in this specific cohort.

### 2.2. Blood Volume Estimation in DBS Samples

#### Determination of Blood Volume in DBS Conductivity Method

**Figure 6:**
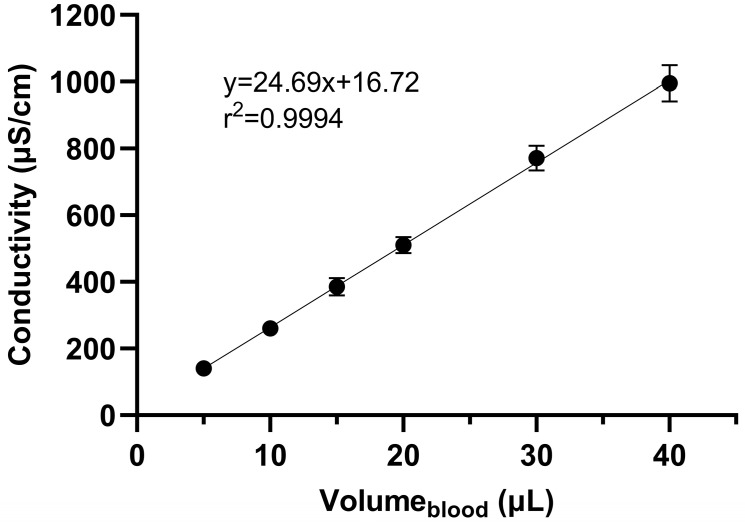
Linear relationship between the volume of DBS and the conductivity detected.

A summary of the validation data for blood volume determination using a 6 mm punch from DBS samples is presented in Table 4. The average curve for HCT values ranging from 0.2 to 0.6 is linear (Figure 6), and QC samples demonstrated appropriate accuracy (96.8–100%) and precision (<13.6%), meeting the defined criteria at all examined levels. The calibration curves obtained at specific HCT values, along with the calculated blood volume for a 6 mm punch using either the average calibration curve or the curve at a specific HCT, are shown in Table 5. Examining the two extreme HCT values (0.2 and 0.6), it is evident that as the HCT increases, the estimated blood volume in the DBS also increases. This is explained by the fact that the relative viscosity of blood increases with higher HCT values. Consequently, a 6 mm punch of a sample with HCT 0.6 contains a higher volume of red blood cells, leading to the estimation of a higher blood volume in the punch. Analysis of covariance (ANCOVA) revealed a significant main effect of the dependent variable conductivity on blood volume, F(1, 140) = 2360.64, *p* < 0.001, indicating that blood volume is a strong predictor of conductivity. The main effect of HCT was not significant, F(4, 140) = 0.90, *p* = 0.464. However, the interaction between blood volume and HCT was significant, F(4, 140) = 7.28, *p* < 0.001, suggesting that the effect of blood volume on conductivity varies across HCT levels. It was confirmed that the calibration curve slope at HCT 0.2 is significantly different from the slope at HCT 0.5 (*p* < 0.001) and HCT 0.6 (*p* < 0.01) (Table 5).

**Figure 7:**
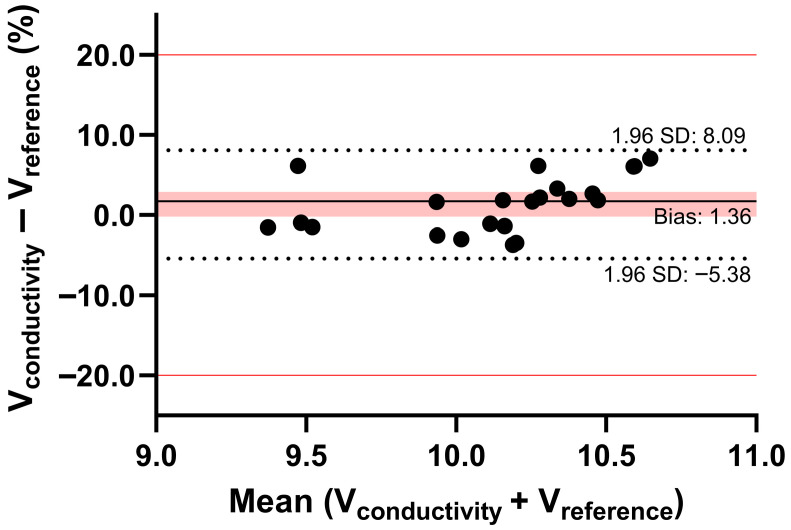
Bland–Altman analysis of estimated blood volumes in the punches of patient DBS samples. Blood volume was determined using the literature method based on surface area and HCT (reference method) and the conductivity method. In the Bland–Altman plot, bias is shown with a solid black line, and the red area indicates the 95% confidence interval. The 95% limits of agreement are indicated with dotted black lines, while a 20% deviation is shown with red lines.

The blood volumes of the DBS sample punches obtained from patients were determined using the conductivity method and the average calibration curve, as the HCT values ranged from 0.35 to 0.46. These blood volumes were compared with those calculated using the reference method developed by Alsous et al. [11]. Alsous et al. [11] developed a model relating surface area (SA) to blood volume (BV) and hematocrit: SA = (690.414 × BV) − (72.3 × HCT%) + 3941.8. The estimation of blood volume on a 6 mm punch from patient DBS samples was obtained by using the surface area (constant value 7780) and applying this relationship. The calculated blood volumes served as the reference. Bland–Altman analysis showed good agreement, with a deviation of less than 10% from the reference values, confirming that our method is reliable and suitable for estimating blood volume in DBS samples (Figure 7). An MPPE of 1.85% and MAPE of 2.49% further support the comparability between the conductivity method and the published method by Alsous et al. for estimating blood volume in a DBS sample [11].

### 2.3. Evaluation of Correction Strategies for HCT and Volume Bias in DBS Analyte Quantification

**Figure 8:**
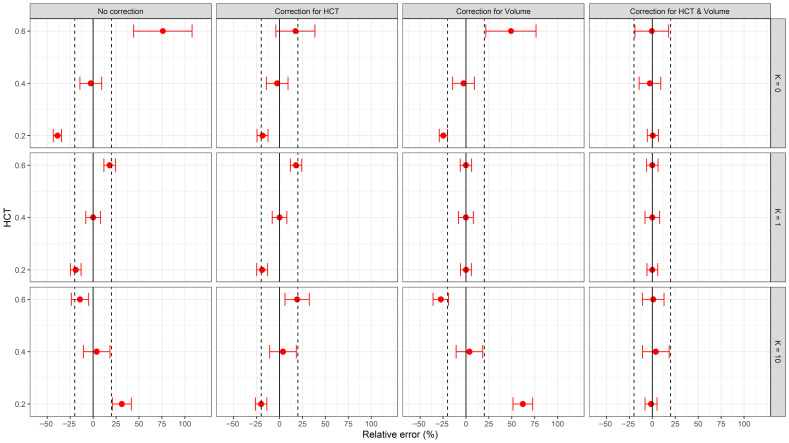
Predicted relative error of calculated drug plasma concentration with 95% uncertainty (error bars) based on measured drug concentration in DBS at various hematocrit (HCT) levels and different red blood cell to plasma partition coefficients (K), without correction and with correction for HCT, volume, or both HCT and volume. The hemoglobin method was used for HCT correction. Extreme values of HCT (0.2 and 0.6) and K (0 for no partitioning in red blood cells, 10 for accumulation in red blood cells) were selected. Solid vertical lines represent the no-effect line (relative error of 0%), and dashed vertical lines indicate a critical error (greater than 20% or less than −20%).

Analyte quantification from DBS samples is influenced by the HCT and blood volume. Variations in HCT and blood volume affect spot morphology, blood viscosity, and analyte partitioning, each of which introduces bias in analyte estimation. Figure 8 shows the predicted relative error in the plasma drug concentration derived from the quantification of various DBS analytes with different partition coefficients (K) at different HCT values. Extreme values of HCT (0.2 and 0.6) and K (0 for no red blood cell partitioning, 10 for accumulation in red blood cells) were selected to represent physiologically relevant limits.

The uncorrected plasma concentrations, shown in the leftmost column of Figure 8, resulted in substantial deviations from the nominal plasma concentrations, especially at the extreme values of HCT and K. Correction for either HCT or volume (middle columns) partially reduced the error, but the residual error remained, except when the sample was corrected for volume and the analyte K was equal to 1. The combined correction for both HCT and volume (rightmost column) was the most robust approach, with relative error values close to 0 and within the ±20% threshold across all scenarios.

These findings highlight the systematic bias in analyte quantification from DBS samples resulting from uncorrected HCT and unknown sample volume. Correction of both parameters is necessary when estimating plasma concentrations from DBS, even in the absence of red blood cell partitioning (K = 0), particularly when the HCT is near the lower or upper limit. When the HCT is close to the average population value (0.4), the results are more promising, as most cases fall within the ±20% threshold, even without correction.

## 3. Discussion

Our research aimed to propose an approach to address issues associated with HCT bias, which is a common challenge in using DBS. HCT is an inter-patient variable that can quantitatively influence DBS analysis [12,13]. We evaluated different approaches and derived an optimal procedure for HCT bias correction, which combines several methods. These methods have been previously reported and applied; however, to our knowledge, this is the first study to integrate image analysis, Hb quantification, and conductivity-based volume estimation into a combined HCT bias correction workflow. This combination offers flexibility and robustness in various DBS sample scenarios.

The easiest and fastest approach is to scan the DBSs. This non-destructive method requires only a scanning device and Fiji software and can be performed in a laboratory or in remote areas where reagents or resources are not readily available. The results from the image analysis can be processed, and the HCT of the patient can be estimated from the blood sample on the DBS card, as described by Del Ben et al. [14]. The simplicity, speed, accuracy, and precision of this method are comparable to those of other techniques published for HCT estimation in DBS samples [15,16,17,18]. The obtained polynomial model was accurate and precise. When comparing the literature model [14] with the model developed in this study, both are interchangeable with the reference method. Notably, the two models were developed across different HCT ranges (0.16–0.48 in the reference model versus 0.2–0.6 in our study). While the literature model shows tighter agreement and minimal bias, the model developed in this study demonstrates acceptable but less precise concordance. Using this relationship, the HCT of a DBS sample can be determined. In our case, the DBSs were scanned within the first three days after sample preparation was completed. Problems may arise as they continue to dry, as their color darkens due to oxidation, eventually turning darker brown. In such cases, other methods can be used. Del Ben et al. [14], to assess the robustness of their model, dried DBS samples at RT for up to nine days and additionally exposed them to neon light and sunlight throughout. Environmental lighting and paper type are important factors that must be evaluated before method application, as they can affect the quality of DBS samples.

When image-based estimation is compromised by oxidation or delayed scanning, a destructive yet highly stable alternative is Hb quantification using SLS, as presented by Richardson et al. [19]. This standardized method enables a direct relationship with HCT based on the Hb/HCT ratio. This method is based on the fact that SLS can form a complex with Hb. The addition of SLS leads to the conversion of oxyhemoglobin, deoxyhemoglobin, carboxyhemoglobin, and methemoglobin to sulfated Hb derivatives. This conversion is rapid, and the end product, the SLS-Hb complex, is stable for a few hours at RT [20] and can be measured using a UV-VIS spectrometer at 550 nm. The influence of parameters known to affect the results, such as the spotted volume of DBS, punch location, and storage time and conditions, was also assessed by Richardson et al. [19]. It was demonstrated that the samples were stable after storage at 4 °C for up to six months (<10% change). This excellent long-term stability is one of the main advantages of this method. In the present study, we also investigated different time intervals for the reaction of SLS with Hb and its derivatives. The ratio of the UV-VIS response for Hb measurement from DBS punches extracted for 10 and 20 min was 1.0; therefore, we selected the method with the shorter extraction time. Additionally, we used the obtained equation to back-calculate the HCT for DBS punches taken from spots with higher volumes (30 and 40 μL). In this case, the HCT was determined with accuracy ranging from 95.7 to 102.2%. This highlights the importance of a universally standardized sampling volume method, as deviations could affect the accuracy of HCT estimation if not properly considered. The Hb method is best suited for clinical settings, as the equipment and reagents are readily available.

The last method tested was conductivity, which was used to determine blood volume in a DBS sample. The first application of the conductivity method for estimating the blood volume in a DBS was performed by Kadjo et al. [21]. The method was developed based on the fact that electrolyte concentrations in blood are tightly regulated. For example, in 99.5% of over 111,000 blood samples analyzed, the concentration of sodium, one of the most prevalent blood ions, was within the range of 120–150 mM [22]. Additionally, sodium ion concentration is higher than that of other blood ions; however, chloride ions have 50% higher mobility, making chloride the electrolyte with the greatest impact on conductivity measurement. This method can significantly reduce errors caused by variations in the spotted blood volume and area of the DBS. It is a rapid method that requires only the addition of ultrapure water to the DBS punch. The instrumentation is suitable for aqueous samples with mid-to-high conductivity. Organic solvents typically contain few free ions and therefore exhibit lower conductivity than aqueous solutions. This parameter must be considered.

During method development, we analyzed the effect of the paper on DBS in two ways. First, we evaluated the response of the benchtop conductivity meter when freshly prepared blood was added directly to ultrapure water, omitting the filter paper but following the same extraction protocol. Second, we simulated the area covered by blood at different volumes (5–40 μL) on a paper card, excised the corresponding area, and measured the conductivity. The presence of the paper affected conductivity by less than 4%, compared to measurements without paper. Additionally, we compared conductivity measurements between freshly prepared DBS cards and those stored at room temperature for up to two weeks. The resulting ratio was 0.98, indicating that room temperature storage for up to two weeks has a negligible effect. We also used the obtained equations to back-calculate the volume of a 20 μL DBS punch. The lower the HCT of the sample, the lower the calculated blood volume on the DBS punch. For example, a 6 mm punch from a 20 μL spot with HCT 0.2 was calculated to be 8.4 μL using the average calibration curve, while a punch from a 20 μL spot with HCT 0.6 was estimated to be 11.4 μL. This was explained by differences in blood viscosity affecting the adsorption by the filter paper and the size of the DBS.

Finally, we used Li-heparin vacutainers instead of ethylenediaminetetraacetic acid (K2-EDTA) vacutainers. This choice was because K2-EDTA vacutainers could influence conductivity measurement results. It has been reported that concentrations of calcium, magnesium, potassium, and chloride can be biased even when blood samples are slightly contaminated with K2-EDTA [23]. As with the Hb method, measuring the conductivity of a DBS sample requires specialized conductivity sensors, but it is important when analyte concentrations need to be normalized based on sample volume. Table 6 summarizes the advantages, disadvantages, and recommendations for when to use the presented methods.

**Figure 9:**
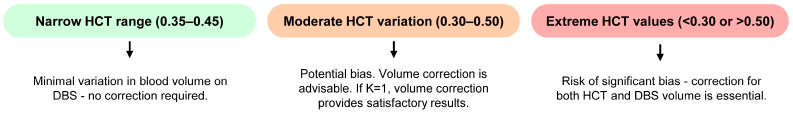
DBS analysis—HCT and volume correction workflow.

In a recently published study [24], we developed, validated, and applied a DBS method for determining UST in patients with IBD. The relatively narrow HCT range observed in that study (0.35–0.46) led to the assumption that HCT would not significantly influence the blood volume collected after a 6 mm punch. Considering the findings of this study, we can retrospectively evaluate the validity of that assumption and quantify the potential impact of extreme HCT values on UST concentration estimates. Based on the results in Figure 8, when the HCT of a study population is close to 0.4 and calibrators with HCT 0.4 are used, correction for either HCT or blood volume of the DBS sample is not necessary. This observation strengthens the robustness of the original approach under specific HCT conditions and establishes a guideline for future applications regarding when correction of DBS samples for HCT and/or blood volume should be implemented. A summary of the findings of this study and a decision workflow for the DBS analysis can be seen in Figure 9.

A limitation of the study was the relatively small dataset, as it included only 23 samples from patients diagnosed with IBD. While the preliminary results are promising, this limits the generalizability of our findings. In addition to the direct effect of HCT on blood viscosity, various acute or chronic inflammatory conditions may exhibit unique blood rheology due to increased blood viscosity [25], which could influence DBS spreading. Therefore, the study’s findings may not be directly applicable to the healthy population or patients with other disorders. It is important for future research to include larger and more diverse demographics to strengthen the evidence and confirm the conclusions of the current study. This could further support the development of a universal HCT-independent volume estimation model.

Additionally, in this study, volume estimation using the conductivity method shows HCT-dependent slope differences. The significant interaction between blood volume and HCT, F(4, 140) = 7.28, *p* < 0.0001, found by ANCOVA, statistically confirms that using the average calibration curve can be limited. This is explained by the increased relative viscosity of blood at higher HCT levels, which affects spreading. The average calibration was effectively used in this cohort, in which DBS samples from IBD patients with a narrow HCT range (0.35–0.46) were obtained. However, the significant interaction between HCT and slope in the analysis of the relationship between conductivity and blood volume at extreme HCT values highlights a limitation for the method’s robustness. In populations where HCT can vary significantly, using the average curve without a specific HCT correction factor could lead to overestimation or underestimation of blood volume. This limitation suggests a pathway for future studies that include such demographic variability. Implementing an additional step for the HCT estimation using image analysis or Hb determination to dynamically select the corresponding calibration curve could reduce errors deriving from average calibration curve.

Another methodological limitation of the research was the exclusive use of 6 mm punches obtained from the DBS samples, which may not represent the variability seen in different methods. Although 6 mm punches are commonly used in bioanalysis, some studies use smaller (e.g., 3.2 mm) punches depending on sensitivity, spot homogeneity, and extraction efficiency. This variability in punch size can affect the volume of blood sampled and, subsequently, the quantification results. Therefore, the choice of punch diameter is an important pre-analytical variable that needs thorough investigation, as it can affect interstudy comparability and the interpretation of DBS concentration estimates.

## 4. Materials and Methods

### 4.1. Chemicals and Materials

Whatman™ 903 Protein Saver Cards (GE Healthcare, Dassel, Germany) were used to collect DBS samples. Venous blood was used to prepare DBS calibrators and quality control (QC) samples. A stainless-steel 6 mm hole puncher (Rayher Hobby & Art, Laupheim, Germany) was used to punch the DBS samples. Ultrapure water was obtained using an A10 Advantage Milli-Q water purification system (Millipore Corp., Billerica, MA, USA). Sodium lauryl sulfate (SLS), supplied by Merck KGaA (Darmstadt, Germany), was also used.

### 4.2. Preparation of DBS Calibrators and QC Samples

DBS samples were prepared by collecting drug-free venous blood in 6 mL lithium heparin (Li-heparin) spray-coated BD Vacutainers^®^ (Beckton Dickinson, Oxford, UK) from five healthy volunteers, which were used to prepare the DBS calibrators or QC samples. Blood with HCT values of 0.2, 0.3, 0.4, 0.5, and 0.6 was prepared from each donor for every experiment, using previously validated methods [26]. A fixed volume of 20 μL drug-free venous blood was applied to Whatman™ 903 filter paper DBS cards, which were then used for image-based HCT estimation experiments. For hemoglobin (Hb) measurement, a 6 mm punch was collected from the 20 μL DBS samples. For the conductivity experiments, DBS samples were prepared by pipetting predetermined volumes of blood (5, 10, 20, 30, and 40 μL) onto the paper cards. In all cases, the DBS samples were dried for at least 3 h, protected from sunlight, and then placed in an airtight resealable plastic bag with a desiccant (Merck KGaA, Darmstadt, Germany) and stored at room temperature until analysis.

### 4.3. Hematocrit Measurement in DBS Samples

#### 4.3.1. Hematocrit Measurement in DBS Samples Using Image Analysis

In this method, 20 μL of blood covering the predetermined HCT range (0.2–0.6) was volumetrically applied on DBS cards. After drying, the cards were scanned using a commercial document scanner (Konica Minolta KM-C368, Konica Minolta Inc., Tokyo, Japan). The DBS scans were saved for further processing in Tagged Image File Format (TIFF). During image processing (ImageJ^®^ software, version 1.54p), the color of the DBS was analyzed by selecting individual spots on the images using the wand tracing tool. The mean gray value (MGV) of the DBS was measured. The MGV is the average gray value within the selected area and represents the sum of the gray values of all pixels in the selection divided by the number of pixels. It ranges from 0 (black) to 255 (white). Based on the results, a polynomial relationship between HCT and MGV was obtained, with the independent variable being the known HCT value and the dependent variable being the intensity of the spot color. Subsequently, HCT of the patient DBS samples was estimated by applying the polynomial relationship between the MGV and HCT.

#### 4.3.2. Hematocrit Determination Using Hemoglobin Measurement by UV-VIS Spectrometry

A similar approach to that presented by Richardson et al. [19] was used to measure Hb in our laboratory. After obtaining a 6 mm punch from the DBS samples (HCT range 0.2–0.6), a 30 min extraction with 100 μL Milli-Q water was performed. Next, 100 μL of SLS was added. Instead of the commercially available sulfolyser, we prepared a 0.17% (*w*/*v*) SLS solution in Milli-Q water. After the addition of SLS, the samples were incubated for 10 min before measuring absorbance at 550 nm using a Tecan Safire 2 (Tecan, Männedorf, Switzerland). The linear equation from the calibration curve was used to calculate the HCT value of the patient samples.

### 4.4. Blood Volume Determination Using the Conductivity Method

A similar methodology was used in our study with some modifications, based on the principle described by Kadjo et al. [21]. To establish the relationship between the volume of blood on a DBS and conductivity, a benchtop conductivity meter FiveEasy FP30 equipped with the pH Sensor InLab^®^ Expert Pro (Mettler Toledo, Greifensee, Switzerland) was used. This device allows measurement of small sample volumes and can measure in the conductivity range of 0.01–100 µS/cm. The conductivity meter was calibrated according to the manufacturer’s instructions. Each DBS sample, covering the HCT range of 0.2–0.6 and applied blood volumes of 5–40 μL, was excised in its entirety and transferred into an Eppendorf tube containing 500 μL of Milli-Q water. The samples were then extracted for 20 min at room temperature. Before measuring conductivity, the Eppendorf tube was briefly vortexed (for 5–10 s).

### 4.5. Validation

For each method, a calibration curve was calculated using a linear or polynomial model. The back-calculated concentrations of the calibrators had to be within ±15% of the nominal values, and the lower limit of quantification (LLOQ) had to be within ±20%. Accuracy and precision were evaluated at three QC levels (low, QCL; medium, QCM; and high, QCH). Accuracy was expected to fall within 85–115% of the nominal value, while precision, expressed as relative standard deviation (RSD), was required to remain below 15%.

### 4.6. Patient DBS Samples

The samples analyzed in this study were obtained from ten adult patients diagnosed with inflammatory bowel disease (IBD) and five healthy volunteers recruited for the prospective observational study, which was approved by the National Medical Ethics Committee of the Republic of Slovenia (0120-013/2016-2, KME 38/01/16) and conducted in accordance with the Declaration of Helsinki. Patients receiving therapy with the monoclonal antibody ustekinumab (UST) were recruited during scheduled outpatient visits at the Department of Gastroenterology, University Medical Centre Ljubljana. In total, 23 samples were analyzed using the methods applied in this study. The HCT reference values were determined at the University Medical Centre Ljubljana using a Microsemi CRP hematology analyzer (Kyoto, Japan).

### 4.7. Data Analysis

Microsoft Excel (version 2019 (16.0), Microsoft Corporation), GraphPad Prism (version 10.0.0, GraphPad Software, Boston, MA, USA) and IBM SPSS Statistics for Windows (version 29.0, IBM Corp., Armonk, NY, USA) were used for data handling, plotting, and statistical analysis. To determine the surface area and HCT of the DBS, images were processed with Fiji, an ImageJ-based platform (version 1.54p) [27]. The relationships between the different methods were assessed using Deming regression and Bland–Altman analysis. For Deming regression, the regression line and 95% confidence intervals were calculated. For Bland–Altman analysis, the mean bias and 95% limits of agreement were calculated to assess systematic differences. The predictive performance of the methods was evaluated by calculating the mean predictive percent error (MPPE) and mean absolute percentage error (MAPE). ANCOVA was used to assess how HCT influences the slope and intercept of calibration curves relating conductivity to blood volume by testing both main effects and their interaction. Calibration curves at specific HCT values were compared to the calibration curve obtained at HCT 0.2. Statistical significance was set at *p* < 0.05. The following equation was used to calculate drug concentration in blood plasma (*Cp*):

$$C_{p}=\frac{C_{b}}{\left(1-HCT\right)+K\;\mathrm{HCT}}\frac{V_{pred}}{V_{nom}}$$

where *Cb* is the measured drug concentration in blood (DBS sample), HCT is the measured hematocrit, K is the blood cell-to-plasma partition coefficient of the drug, and *Vpred* and *Vnom* are the predicted and nominal volumes of the blood sample, respectively. The error in the calculated drug plasma concentration relative to the typical HCT value of 0.4 and its uncertainty were calculated in R software (version 4.5.2, R Core Team, https://www.R-project.org) using Monte Carlo simulation of the above equation. Inputs (HCT and *Vpred*) were defined as normal distributions, considering the accuracy and precision of these determinations. Vectors of 100,000 random numbers were generated from the distributions of HCT and *Vpred* using the *rnorm()* function, and each time, the drug concentration in blood plasma (*Cp*) was calculated for three hypothetical drugs with K values of 0, 1, and 10, assuming no distribution in blood cells, equal distribution between blood cells and plasma, and accumulation in blood cells, respectively. Relative errors of *Cp* were then calculated as medians of the generated distributions, and their uncertainties were calculated as the 2.5th and 97.5th percentiles.

## 5. Conclusions

We successfully measured and estimated HCT in DBS samples using two different methods (image analysis and Hb determination) and the blood volume of DBS (conductivity) by applying three different methods accurately and precisely. Application of the developed methods to patient DBS samples showed that the methods accurately determine HCT and blood volume with acceptable deviations from the reference methods. Therefore, the presented methods provide an appropriate tool to enable quantitative measurement of analyte concentration in non-volumetric DBS samples in both clinical diagnostics and research, supporting ongoing efforts to standardize DBS-based quantification across diverse clinical settings. They offer solutions to some limitations of DBS analysis, potentially improving the accuracy and consistency of the DBS sampling method.

## Figures and Tables

**Table 1:** Validation data for HCT determination using image analysis and MGV.

Method Range	Calibration Curve (*n* = 10)	r^2^	Level (*n* = 5)	Nominal	Accuracy (%)	Precision (%)
0.20–0.60	MGV = 161.67 × HCT^2^ − 237.12 × HCT + 177.64	0.998	QC_L_	0.20	90.3	0.87
			QC_M_	0.40	93.2	2.71
			QC_H_	0.60	98.8	4.77

MGV—mean gray value; HCT—hematocrit; QC_L_, QC_M_, QC_H_—quality control samples at low, medium and high concentration, respectively; r^2^—coefficient of determination; *n*—number of samples.

**Table 2:** Validation data for HCT determination using image analysis of surface area.

Method Range	Calibration Curve (*n* = 10)	r^2^	Level	Nominal (*n* = 5)	Accuracy (%)	Precision (%)
0.20–0.60	SA = −0.0397 × HCT + 0.0922	0.998	QC_L_	0.20	103	11.3
			QC_M_	0.40	103	5.73
			QC_H_	0.60	99.6	5.63

SA—surface area; HCT—hematocrit; QC_L_, QC_M_, QC_H_—quality control samples at low, medium and high concentration, respectively; r^2^—coefficient of determination; *n*—number of samples.

**Table 3:** Validation data for HCT determination using hemoglobin method.

Method Range	Calibration Curve (*n* = 5)	r^2^	Level (*n* = 5)	Nominal	Accuracy (%)	Precision (%)
0.20–0.60	ABS = 1.137 × HCT + 0.003	0.9939	QC_L_	0.20	102.2	2.45
			QC_M_	0.40	95.7	7.44
			QC_H_	0.60	99.1	5.87

ABS—absorbance at 550 nm; HCT—hematocrit; QC_L_, QC_M_, QC_H_—quality control samples at low, medium and high concentration, respectively; r^2^—coefficient of determination; *n*—number of samples.

**Table 4:** Validation data for blood volume determination in DBS samples using the conductivity method.

Method Range	Calibration Curve (*n* = 5)	r^2^	Level (n = 5)	Nominal	Accuracy (%)	Precision (%)
5.0–40.0	Vol = 24.7 × Cond + 16.7	0.999	QC_L_	0.20	96.8	13.6
			QC_M_	0.40	100	5.57
			QC_H_	0.60	99.6	5.14

Vol—blood volume (µL); Cond—conductivity (µS/cm); QC_L_, QC_M_, QC_H_—quality control samples at low, medium and high concentration, respectively; r^2^—coefficient of determination; *n*—number of samples.

**Table 5:** Relationship between blood volume and conductivity of DBS extracts, in addition to the estimated blood volumes of DBS punches for DBS samples prepared with different blood HCT.

Parameter	HCT 0.2	HCT 0.3	HCT 0.4	HCT 0.5 *	HCT 0.6 *	HCT 0.2–0.6
C**alibration Curve**						
Slope	25.97	26.06	25.18	23.01	23.22	24.69
Intercept	10.19	13.15	−0.01	29.78	27.60	16.72
r^2^	0.997	0.999	0.999	0.996	0.997	0.999
**Blood volume (μL)**						
HCT-specific calibration curve ^1^	8.1 ± 0.29	9.1 ± 0.27	10.1 ± 0.38	11.0 ± 0.32	11.7 ± 0.32	–
HCT 0.2–0.6 calibration curve ^2^	8.4 ± 0.31	9.5 ± 0.29	9.6 ± 0.39	10.5 ± 0.32	11.4 ± 0.30	–

^1^ 6 mm punch obtained from 20 μL DBS; volume of blood calculated using calibration curve from calibrators with the specific HCT. The average blood volume and its standard deviation are indicated. ^2^ 6 mm punch obtained from 20 μL DBS; volume of blood calculated using average calibration curve from calibrators with HCT 0.2–0.6. The average blood volume and its standard deviation are indicated. * ANCOVA results: slope of calibration curve at specific HCT is significantly different from the slope of calibration curve at HCT 0.2. r^2^—coefficient of determination. HCT—hematocrit.

**Table 6:** Summary table for the presented methods.

Method	Target	Advantages	Disadvantages	When to Use
**Image analysis method**	Hematocrit determination	- Non-destructive, no reagents required - Rapid estimation directly from DBS - Suitable for field or low-resource settings	- Needs imaging device/software - Accuracy influenced by spot quality, lighting, or paper type	- Preferred in remote areas, large-scale screenings, or when reagent-free estimation is needed
**Hemoglobin method**	Hematocrit determination	- Well-established and standardized - Direct relation to HCT through Hb/HCT ratio - Widely available in clinical labs	- Destructive - Requires reagents/instruments - Sensitive to sample degradation	- Best for routine, high-accuracy clinical settings where equipment is available
**Conductivity method**	DBS volume estimation	- Direct physical measurement of blood spot volume - No biochemical interference - Useful for normalizing analyte concentrations	- Destructive - Requires specialized conductivity sensors - Sensitive to humidity and substrate variations - Less commonly available in labs	- Suitable when accurate DBS volume estimation is critical for quantitative assays

## Data Availability

The original contributions presented in this study are included in the article. Further inquiries can be directed to the corresponding author.

## Abbreviations

The following abbreviations are used in this manuscript:

ANCOVA	Analysis of covariance
95% CI	95% Confidence Interval
*Cb*	Drug concentration in blood
*Cp*	Drug concentration in blood plasma
DBS	Dried blood spots
Hb	Hemoglobin
HCT	Hematocrit
IBD	Inflammatory bowel disease
K	Drug blood cell-to-plasma partition coefficient
K2-EDTA	Ethylenediaminetetraacetic acid
Li-heparin	Lithium heparin
LLOQ	Lower limit of quantification
LoA	Limits of agreement
MAPE	Mean absolute percentage error
MGV	Mean gray value
MPPE	Mean predictive percent error
QC	Quality control
QCH	Quality control high concentration
QCL	Quality control low concentration
QCM	Quality control medium concentration
RSD	Relative standard deviation
SLS	Sodium lauryl sulphate
TDM	Therapeutic drug monitoring
TIFF	Tagged image file format
UST	Ustekinumab
UV-VIS	Ultraviolet–visible spectrophotometry
*V_pred_*	Predicted volume of blood sample
*V_nom_*	Nominal volume of blood sample
